# MEANINGS OF AGING IN THE RIGHT PLACE FOR HOUSING INSECURE OLDER ADULTS IN TEMPORARY HOUSING

**DOI:** 10.1093/geroni/igad104.0207

**Published:** 2023-12-21

**Authors:** Atiya Mahmood, Sarah Canham, Rachel Weldrick, Rachelle Patille, Morgan Erisman

**Affiliations:** Simon Fraser University, Vancouver, British Columbia, Canada; University of Utah, Salt Lake City, Utah, United States; Simon Fraser University, Vancouver, British Columbia, Canada; Simon Fraser University, Vancouver, British Columbia, Canada; University of Utah, Salt Lake City, Utah, United States

## Abstract

The concept of aging in place (AIP) is widely acknowledged as the preference of older adults. However, AIP without consideration of the intersections of diversity in later life is critiqued as lacking an equity lens. This gave rise to the exploration of what is needed to age in the right place (AIRP). Building on existing research, we examined the meaning of AIRP to older adults (aged 55+ years) who have experienced homelessness. We conducted photovoice interviews with 11 residents of a temporary housing program in Metro Vancouver (Canada). Using thematic analysis, we investigated the meanings of AIRP based on 1) How one feels, 2) What one does, and 3) Where one lives. Findings demonstrate that meanings of AIRP follow a similar pattern as Maslow’s Hierarchy of Needs. That is, when basic needs of shelter are met, participants’ considerations of the ‘right’ place to live extends beyond affordable housing to include having access to meaningful activities – in urban districts and nature-scapes – enabled by affordable transportation. This consideration includes higher-order factors such as opportunities for meaningful engagement and looking forward to a stable life, being at peace, feeling safe and comfortable in one’s home and neighborhood. Our examination of what AIRP means to this group of older adults broadens current conceptualizations. Given the increase of older adults who are homeless it is imperative that policymakers and practitioners be cognizant of meanings of AIRP to enable diverse groups of older adults to thrive in housing that is more than a shelter.

